# Alpha-kinase 1 agonists derived from microbial metabolite inflame antitumour immunity

**DOI:** 10.1186/s43556-026-00452-1

**Published:** 2026-05-16

**Authors:** Shilei Qiao, Zhengkui Zhang, Fangfang Zhou

**Affiliations:** 1 The First Affiliated Hospital, the Institutes of Biology and Medical Sciences, Suzhou Medical College, Soochow University, Suzhou, Jiangsu, China; 2 MOE Key Laboratory of Geriatric Disease and Immunology, Soochow University, Suzhou, Jiangsu China; 3 Biomedical Basic Research Center of Jiangsu, Soochow University, SuzhouJiangsu, 215123 China

In a recent study published in *Nature* [1], Tian et al. revealed for the first time that novel agonists targeting the cytosolic bacterial receptor α-kinase 1 (ALPK1) induce highly efficient antitumour immunity. These agonists demonstrate a significant advantage over Toll-like receptor (TLR) and interferon gene stimulating factor (STING) agonists, showcasing a powerful targeted strategy for cancer immunotherapy [1].

Due to the insufficient response rate of immune checkpoint blockades (ICBs) therapy, robust antitumour immunity may require an appropriate level of proinflammatory response, which may depend on the activation of innate immunity [2]. Accordingly, innate immune agonists (such as STING or TLR agonists) have emerged to complement ICBs, but their clinical translation is hampered by several limitations, including systemic toxicity, immunosuppression, and T-cell apoptosis and restricted expression of their cognate receptors on immune cells [3]. These challenges reflect broader issues of immune evasion and complex regulatory networks in the tumor microenvironment (TME) [2]. ALPK1 offers a distinct advantage: it is broadly expressed across both immune and non-immune cells and directly promotes antigen presentation, making it well-suited to remodel the immunosuppressive TME.

In 2018, the same research group identified ALPK1 as the pattern recognition receptor for the bacterial metabolite ADP-heptose (ADP-Hep), establishing its critical role in host antibacterial immunity. Upon ADP-Hep binding, ALPK1 phosphorylates TIFA (TRAF-interacting protein with forkhead-associated domain) to activate the NF-κB signaling pathway [4]. However, its role in antitumour immunity remained unelucidated. In this study, the authors further demonstrated that ADP-Hep and its potent derivative UDSP-Hep activate ALPK1, triggering robust innate immune responses, inhibiting the growth of multiple mouse tumour models, and synergizing with ICBs. Moreover, this study systematically evaluated the mechanisms underlying ALPK1 agonist-mediated antitumour immunity, including immune‑activating profiles, sensitivity to ALPK1 polymorphisms across mouse strains, and capacity to reshape the TME (Fig. 1a, b).

**Fig. 1:**
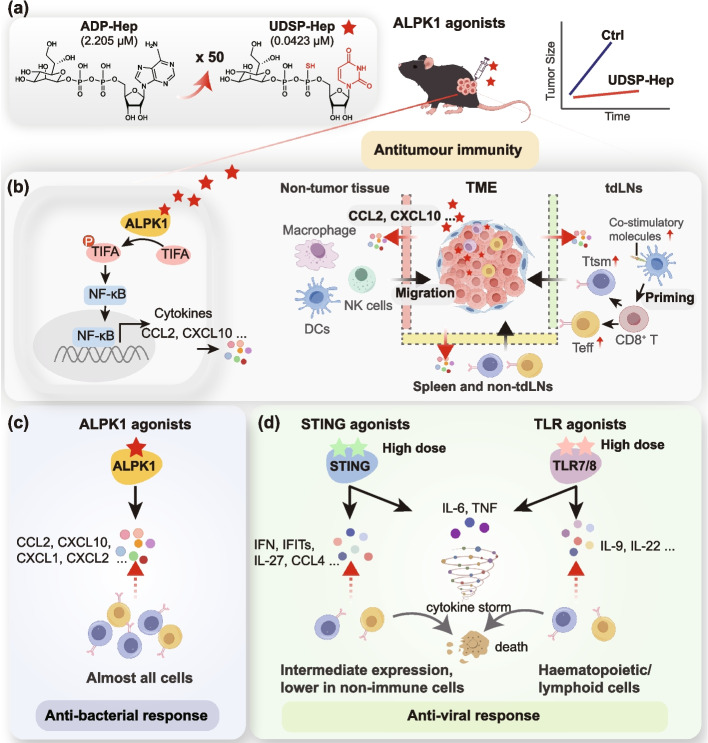
ALPK1 agonists induce highly efficient antitumour immunity. **a** UDSP-Hep, a phosphorothioate analog of ADP-Hep, has an approximately 50-fold increased ability to activate ALPK1. Intratumoral injection of those ALPK1 agonists significantly inhibited tumour growth and induce efficient antitumour immunity. **b** After intratumoral injection, ALPK1 agonists activate ALPK1 to phosphorylate TIFA, then initiating NF-κB signaling pathway, increasing the release of antitumour cytokines, particularly CCL2 and CXCL10. The agonists systematically reshape antitumour immunity (including TME, adjacent non-tumour tissue, tdLNs, and spleen/non- tdLNs), promoting the function and infiltration of tumor-specific CD8^+^ T cells. Specifically, UDSP-Hep enhances cDC1-mediated cross-presenting antigens to promote the differentiation of CD8^+^ T cells into tumor-specific CD8^+^ Teff and Ttsm cells in tdLNs, which subsequently infiltrate into tumour and exert durable anti-tumour functions and generate long-lasting immune memory. **c**, **d** Compared to STING/TLR agonists, ALPK1 agonists have unique mechanisms and advantages: (1) ALPK1 is widely expressed in almost all cells (including non-immune cells), while the expression of STING or TLRs is mainly limited to immune cells. (2) Low doses of ALPK1 agonists, especially UDSP-Hep, can significantly inhibit tumour growth, while STING/TLR agonists require high doses to achieve similar effects. (3) ALPK1 agonists specifically activate an anti-bacterial immune response, while TLR/SITNG agonists activate an anti-viral immune response. There are distinct transcriptional and cytokine signatures between them. ALPK1 agonists induce the expression of chemokines (such as CCL2, CXCL10, CCL4 and CCL9), thereby effectively promoting the function and infiltration of tumor-specific CD8^+^ T cells. However, TLR/SITNG agonists not only exhibit weak chemotactic ability but also specifically induce IL-6 and TNF, which may lead to a cytokine storm, triggering T cell apoptosis/exhaustion. Abbreviation: ADP-Heptose, ADP-Hep; UDSP-Heptose, UDSP-Hep; Alpha kinase 1, ALPK1; tumor microenvironment, TME; tumor-draining lymph nodes, tdLNs; cDC1, conventional type 1 dendritic cells; memory CD8^+^ T cells, CD8^+^ Ttsm; effector CD8^+^ T cells, CD8^+^ Teff

Intratumoral injection of ADP-Hep significantly inhibited tumour growth across multiple syngeneic mouse tumour models. In *Alpk1*^*−/−*^ mice, the antitumour effect was abrogated, whereas mice carrying a gain-of-function ALPK1(T237M) mutation exhibited enhanced tumour suppression, indicating that the antitumour efficacy depends on host ALPK1 signaling.

Through medicinal chemistry optimization, a phosphorothioate analog of ADP-Hep, designated UDSP-Hep, was developed. This compound exhibits approximately 50-fold higher potency in activating ALPK1(as measured by EC_50_) than the parent molecule ADP-Hep, and elicits robust pro-inflammatory cytokine secretion and stronger adaptive immunity, and these effects are strictly ALPK1-dependent (Fig. 1a). UDSP-Hep also displays selectivity across mouse strains. For example, 129 mice carrying hypomorphic *Alpk1* alleles show weak responsiveness to UDSP-Hep. Notably, mice achieving complete tumour regression after UDSP-Hep treatment were fully resistant to subsequent tumour rechallenge, indicating that ALPK1 activation elicits durable antitumour immune memory. A significant distal effect was also observed, whereby intratumoral UDSP-Hep administration inhibited the growth of untreated, contralateral tumours. Furthermore, UDSP-Hep exerted synergistic effects with multiple ICB agents to enhance their therapeutic efficacy. Collectively, these findings underscore the potent antitumour immune activity and broad translational potential of UDSP-Hep.

Regarding the mechanism of action of UDSP-Hep, the study demonstrated that its antitumour activity depends on CD8^+^ T cells, dendritic cells (DCs), and macrophages, as well as the chemokines, CXCL10 and CCL2. Antibody-mediated blockade of the CXCL10 receptor or direct neutralization of CCL2 completely abrogated the antitumour effect of UDSP-Hep. Similarly, the antitumour activity was lost following administration of a CD8-depleting antibody, surgical resection of tumor-draining lymph nodes (tdLNs), or use of *Batf3*^*−/−*^ mice lacking functional cDC1s. Consistently, CD8^+^ T cells isolated from tdLNs of UDSP-Hep-treated mice exerted potent tumour-controlling activity when adoptively transferred into *CD8α*^*⁻/⁻*^ mice bearing tumours. Mechanistically, UDSP-Hep enhances cDC1-mediated cross-presentation and activation of tumor-specific CD8^+^ T cells. Following UDSP-Hep treatment, expression of co-stimulatory molecules such as CD80, CD86, and CD40 on cDC1s was upregulated, and the proportion of CD69^+^ T cells within the CD8^+^ T cell in the tdLNs was significantly increased. Single-cell RNA sequencing analysis further validated that UDSP-Hep remodels the TME from an immunosuppressive to a pro-inflammatory, antitumour state, increasing the proportions of effector and memory T cells while decreasing exhausted T cells and protumour macrophages (Fig. 1b). However, the article lacks discussion on effects on immunosuppressive cells. Whether this agonist modulates other immunosuppressive components in the TME remains an open question. ALPK1 agonists offer several distinct advantages over STING and TLR agonists. First, ALPK1 and TIFA are widely expressed across 73 cell lines, whereas TLR7/8/9 expression is restricted to hematopoietic and lymphoid cells, and STING expression is relatively low in non-immune cells. Unlike TLR/STING agonists, which primarily target immune cells and lack allele-specific selectivity, UDSP-Hep exhibits a broader cellular target range and is less likely to induce non-specific immune activation. In terms of immunomodulation, UDSP-Hep efficiently activates cDC1s in tdLNs, directly enhances MHC-I antigen presentation by tumour cells, promotes expansion of tumor-specific CD8⁺ T cells and induces macrophage-DC cross-priming without triggering T cell apoptosis. In contrast, TLR agonists exhibit low selectivity for cDC1 and are prone to cause T cell apoptosis and exhaustion [2], while STING agonists only moderately activate DCs and significantly induce T cell apoptosis. Regarding TME remodeling, UDSP-Hep strongly induces CXCL10 and CCL2, promotes effector immune cells infiltration, and upregulates antigen presentation by tumour cells. By comparison, STING agonists display weak chemotactic ability, TLR agonists tend to cause excessive neutrophil infiltration [2], and neither activates tumour cell antigen presentation. Finally, in terms of efficacy and safety, UDSP-Hep outperforms TLR/STING agonists, which exhibit weak single-agent activity, limited combination effects, poor protective immune memory, and a propensity for adverse effects such as systemic inflammation. Specifically, UDSP-Hep effectively inhibits multiple tumour types as a monotherapy, exerts significant synergistic effects with ICBs, induces long-lasting protective memory T cells, and demonstrates low toxicity without overt systemic hyperinflammation, representing distinct therapeutic advantages (Fig. 1c, d).

The ALPK1 small molecule agonist PTT-936 independently developed by this research team, initiated a Phase 1b clinical trial in 2024 (NCT06244992) to evaluate its preliminary efficacy and safety as monotherapy or in combination with anti-PD-1/L1 agents in patients with locally advanced or metastatic solid tumours. In 2025, it was approved by the National Medical Products Administration of China as a Class 1 new drug for clinical research, underscoring the potential clinical applicability of UDSP-Hep. However, several key considerations remain: (1) Possible clinical *ALPK1* genetic polymorphism. The potential impact of *ALPK1* genetic polymorphisms on therapeutic response warrants further investigation, as loss-of-function variants, if present, could affect treatment outcomes; (2) Limitations of the administration route. Intratumoral injection of UDSP-Hep is limited to accessible tumours. For deep-seated or metastatic lesions, emerging drug delivery systems (e.g., nanocarriers, targeted conjugation) may enable systemic administration with enhanced tumour specificity; (3) Long-term safety evaluation. Sustained ALPK1 activation may trigger intestinal inflammation or autoimmune manifestations (e.g., ROSAH syndrome) [5]. Therefore, establishing a clinical dose-efficacy-toxicity correlation model is needed. The breakthrough significance of this research lies in demonstrating that mimicking “bacterial infection” signals (via ALPK1 agonism) can safely and potently activate antitumour immunity, offering distinct advantages over mimicking “viral infection” signals (e.g., STING agonists). ALPK1 agonists exhibit distinct mechanisms and advantages: Firstly, the receptor is more broadly expressed in different cell types, especially in non-immune cells. Secondly, this pathway directly promotes tumour cell antigen presentation and induce protective memory T cell differentiation without causing T cell death. It offers a well-defined target and more promising candidate drug molecules for the development of next-generation intratumoral immunotherapies.

## Data Availability

Not applicable.
